# Water and hemoglobin modulated gelatin-based phantoms to spectrally mimic inflamed tissue in the validation of biomedical techniques and the modeling of microdialysis data

**DOI:** 10.1117/1.JBO.27.7.074712

**Published:** 2022-02-01

**Authors:** Hanna Jonasson, Chris D. Anderson, Rolf B. Saager

**Affiliations:** a Linköping University, Department of Biomedical Engineering, Linköping, Sweden; b Linköping University, Department of Biomedical and Clinical Sciences, Linköping, Sweden

**Keywords:** tissue simulating phantom, water, gelatin, hemoglobin, diffuse optical spectroscopy

## Abstract

**Significance:**

Tissue simulating phantoms are an important part of validating biomedical optical techniques. Tissue pathology in inflammation and oedema involves changes in both water and hemoglobin fractions.

**Aim:**

We present a method to create solid gelatin-based phantoms mimicking inflammation and oedema with adjustable water and hemoglobin fractions.

**Approach:**

One store-bought gelatin and one research grade gelatin were evaluated. Different water fractions were obtained by varying the water-to-gelatin ratio. Ferrous stabilized human hemoglobin or whole human blood was added as absorbers, and the stability and characteristics of each were compared. Intralipid^®^ was used as the scatterer. All phantoms were characterized using spatial frequency domain spectroscopy.

**Results:**

The estimated water fraction varied linearly with expected values ($R^{2}=0.96$ for the store-bought gelatin and $R^{2}=0.99$ for the research grade gelatin). Phantoms including ferrous stabilized hemoglobin stayed stable up to one day but had methemoglobin present at day 0. The phantoms with whole blood remained stable up to 3 days using the store-bought gelatin.

**Conclusions:**

A range of physiological relevant water fractions was obtained for both gelatin types, with the stability of the phantoms including hemoglobin differing between the gelatin type and hemoglobin preparation. These low-cost phantoms can incorporate other water-based chromophores and be fabricated as thin sheets to form multilayered structures.

## 1 Introduction

Translational research involves the *in vivo* study of abnormal or provoked tissue, not least the skin. Two key components of the inflammatory processes often studied are redness (erythema) and swelling (oedema). Cutaneous microdialysis is a minimally invasive technique to enable *in vivo* sampling of endogenous and exogenous substances in the interstitial fluid of the dermis. By inserting a small microdialysis catheter, substances can diffuse from tissue through the membrane of the microdialysis catheter to be collected and analyzed. Microdialysis is used both in clinical research in many organs including skin (percutaneous penetration, pharmacokinetics, skin metabolism, and skin inflammation) and in clinical monitoring in intensive care settings.^1^ However, for optimal interpretation of the high-sensitivity microdialysis data obtained, normalization is required for blood tissue concentration and the degree of swelling (water content) in the target tissue.^2^ Quantification of erythema is relatively easily achieved by non-invasive spectrophotometric techniques such as polarization spectroscopy or by use of an internal calibrator in the perfusate, but there is as yet no robust method for quantification of water in living tissue in real time.^1^^,^^3^^,^^4^ Our group is developing the use of spatial frequency domain spectroscopy (SFDS) to provide real-time data on tissue water content.

Tissue-mimicking phantoms are already an important component in biomedical optics research for system development, calibration, and evaluation. We see an opportunity to improve microdialysis data modeling by extending standard bench top preparatory recovery protocols to phantoms in which the blood and water content can be regulated. Depending on clinical applications, tissue types, and/or desired optical properties, there are various types of phantom matrix options such as an aqueous suspension, gelatin or agar-based matrix, and silicone.^5^ These are mixed with absorbing components such as inks and dyes or whole blood and scattering components such as Intralipid^®^, polystyrene microspheres, and metal oxide powders to mimic the optical properties of the target tissue.^5^

Most skin mimicking phantoms are focused on including absorption from melanin and oxy- and deoxyhemoglobin. Still, there are applications such as monitoring wound healing, inflammation, and oedema in which there is a need to vary the water fraction in addition to hemoglobin in phantoms to mimic tissue pathology in various stages. Additionally, phantoms with varying water fractions are needed to evaluate NIR system performance in estimating water content. Common optical sources (e.g., LEDs) and silicon-based detectors tend to have relatively weaker responses, in terms of their emission intensity or their detection sensitivity, respectively, around the 970-nm band associated with water relative to the shorter wavelength range in the near-infrared and the visible spectra, resulting in low signal to noise (and consequently, a potentially biased estimation of the water fraction).

Phantoms including water and Intralipid^®^ are widely used, and it has been demonstrated that the water fraction can be altered by changing the fat to water ratio.^6^^,^^7^ However, scattering will become inversely linked to the water fraction of the phantom. Decreasing the water fraction by increasing the proportion of lipid droplets in the phantoms also increases the number density of lipid droplets and thus the scattering of the phantom. Furthermore, when increasing the fraction of Intralipid^®^ in the phantoms, the scattering of the phantom becomes very high, which decreases the similarity between the resulting phantom optical properties and the targeted biological tissue.

Silicone is highly hydrophobic, which makes including water in silicone phantoms difficult. However, it has been demonstrated that some infrared dyes can be used to approximate the absorption features of water. Kennedy et al.^8^ showed how a phthalocyanine 9606 dye can be incorporated in silicone phantoms. This offers a phantom with a long lifespan and provides similar absorption features to that of water around 970 nm, but the dye also includes a broad baseline absorption across the visible- and near-infrared ranges that is not present in water itself. This additional absorption feature can be accounted for and poses a minimal impact in many system validation studies limited to the 900- to 1000-nm range. It could, however, impact the optical properties of phantoms for broader spectral ranges and if additional chromophores, such as hemoglobin, are included in the phantoms.

Gelatin phantoms are biocompatible and provide the possibility to alter the water fraction.^9^ Gelatin-based phantoms have been used frequently in ultrasound and magnetic resonance imaging^10^^–^^12^ and optical spectroscopy.^7^^–^^9^^,^^13^ Even though the lifespan is limited, gelatin also allows for the inclusion of water-soluble absorbers and organic molecules and can be molded into a variety of 3D shapes.^5^ Furthermore, gelatin phantoms are inexpensive to produce and offer simple manufacturing processes. However, to maintain the optical properties and reduce the evaporation of water, gelatin phantoms need to be refrigerated and stored in airtight containers.^5^

Powdered hemoglobin,^14^^–^^16^ purified hemoglobin,^17^ and whole blood^18^ have been added to gelatin phantoms with good results. Saiko et al.^14^ used lyophilized human hemoglobin powder to obtain various levels of methemoglobin concentrations in the phantoms. Du Le et al.^17^ used purified hemoglobin in combination with agar gel and yeast to alter the oxygenation level to create low-cost phantoms with good reproducibility. A systematic comparison of these differing forms and preparations of hemoglobin in a gelatin phantom has, however, yet to be presented.

In this paper, we describe a robust method for creating solid gelatin-based phantoms with physiological relevant and adjustable water fractions. Two different gelatin types are evaluated and compared with regard to their intrinsic optical properties and stability over a range of water-to-gelatin ratios. Additionally, a comparison between two preparations of human hemoglobin, i.e., ferrous stabilized hemoglobin powder or whole blood, is also considered in the context of these two different types of gelatins investigated. Here the primary objective is to evaluate the spectral absorption properties and stability of these gelatin phantoms in the context of mimicking tissue oedema and inflammation by varying both water and hemoglobin content, independent from the scattering properties of these phantoms.

## 2 Method

### 2.1 Varying Water Fraction

Phantoms were prepared by mixing gelatin with water and Intralipid^®^ 20%. Two different gelatins were used: one research grade gelatin, 110-Bloom gelatin derived from acid-cured bovine bone (104078 Gelatin powder, Emprove^®^ Essential, Germany), and one store-bought porcine gelatin powder for food production (Torsleffs, Denmark). After mixing gelatin, water, and Intralipid^®^, the mixture was placed on a heating plate and slowly heated to 55°C while being stirred constantly. To remove air bubbles that resulted from the stirring process, the mixture was then placed into a vacuum chamber at 20 kPa for 10 min. Finally, the mixture was poured into 50-ml plastic beakers and placed into a 4°C refrigerator for at least 2 h to allow the mixture to fully solidify.

The absorption coefficient at 970 nm of the phantoms was independently varied by changing the water-to-gelatin ratio of both types of gelatins. Four levels of weight percentage of water were used, ranging from 89% to 40%. The amount of Intralipid^®^ 20% in the phantoms was kept constant at 10%, i.e., 2% Intralipid per total volume (Table 1), and hence the total water weight fractions in the phantoms were 0.97, 0.88, 0.68, and 0.48.

**Table 1:** Weight percentage of water, gelatin, and Intralipid^®^ 20% in the phantoms. Total water fraction assumes 80% water in Intralid^®^ 20%.

Phantom	Water (%)	Gelatin (%)	Intralipid^®^ 20% (%)	Total water fraction
1	89	1	10	0.97
2	80	10	10	0.88
3	60	30	10	0.68
4	40	50	10	0.48

### 2.2 Hemoglobin Phantoms

Ferrous stabilized human hemoglobin (H0267, Sigma-Aldrich, USA) or whole human blood was used to create tissue simulating phantoms with hemoglobin characteristics. The whole blood was collected from a healthy volunteer by trained clinicians at Linköping University Hospital, and heparin was added to prevent coagulation.

The phantoms were prepared by first dissolving the gelatin in the water to avoid heating the hemoglobin or the whole blood used in the hemoglobin phantoms. The water was heated to boiling, and either 2.5 g of store-bought gelatin or 2.5 g of research grade gelatin was slowly added to 25 g of water while the mixture was stirred vigorously. The temperature of the mixture was then maintained above 55°C for about 10 to 15 min to allow the gelatin to dissolve and the mixture to become homogeneous. After the gelatin had dissolved completely, the mixture was left to cool down to ∼37°C. An amount of 40 mg of ferrous stabilized hemoglobin or 0.1 ml of whole human blood was mixed with 3 g of Intralipid^®^ and then gently added to the mixture of water and gelatin. The phantom was then placed into a vacuum chamber at 20 kPa for ∼10  min and after that transferred into 20-ml Petri dishes and placed into a 4°C refrigerator for 2 h.

### 2.3 Characterization

After fabrication, the absorption coefficient $\mu_{a}(\lambda )$ and the reduced scattering coefficient $\mu_{s}^{\prime }(\lambda )$ were measured using SFDS. The SFDS technique has been described previously in detail by Saager et al.^19^ Briefly, sinusoidal patterns with various spatial frequencies and phases are projected on the sample, and the diffuse reflected light from a ∼600-μm spot in a central location is detected by a spectrometer. To calculate the optical properties of the phantoms, a White Monte Carlo inverse solver was employed to fit the measured spatial frequency-dependent reflectance curve.^20^ This approach determines a unique pair of absorption ($\mu_{a}$) and reduced scattering ($\mu_{s}^{\prime }$) coefficients at each individual wavelength, without any *a priori* spectral constraints to either optical property. For these reasons, this technique was selected as the measurement platform to characterize the optical properties of these phantoms, independent of any spectral priors or expectations of the resulting absorption spectra. This technique has also been previously used in several other characterization and evaluation studies of optical phantom fabrication techniques and media.^8^^,^^19^^,^^21^^–^^23^

The in-house built system used for the measurement used a spectrometer with a wavelength range of 450 to 1000 nm and a resolution of 0.5 nm (AvaSpec-ULS2048CL-EVO-VA-50, Avantes BV, The Netherlands) and a Quartz Tungsten Halogen light source (21DC-3AHD-TQB-FILT, Techniquip, CA, USA) coupled to a modified digital micromirror projector unit (AJP-4500 DMD Projector, Ajile Light Industries Inc., Canada) that provided the spatial frequency illumination patterns. Five evenly spaced spatial frequencies ranging from 0 to $0.2\;{\mathrm{mm}}^{-1}$ were used. Crossed polarizers were used in front of both the projector and detection arms of the system to block specular reflections, and the system was calibrated with a reference turbid phantom of known optical properties to account for spectral and imaging characteristics of the instrument.

The absorption spectrum was then fit in a linear least-squares sense to a basis set of tabular absorption spectra for oxygenated and reduced hemoglobin (in relation to micromolar concentration), methemoglobin (micromolar concentration), and water (volume fraction),^24^^,^^25^ together with the independently characterized absorption spectrum of the different gelatin types. Gelatin absorption was estimated based on the absorption coefficient that could not be accounted for by the water absorption spectrum (i.e., the non-negative residual) calculated from SFDS across all ratios of gelatin to water for both gelatin types.

For the phantoms with varying water fraction, three batches of phantoms with four levels of water fraction for each gelatin type were manufactured and multiple measurements were performed on each of the phantoms to evaluate homogeneity of the phantoms. Simple linear regression was used to fit estimated water fractions measured by SFDS to the water fraction used in the fabrication of the phantoms. Stability of the hemoglobin phantoms was evaluated by characterizing the phantoms at several timepoints: after 2 h, 4 h, 1 day, 2 days, 3 days, and 4 days. The phantoms with variable water fractions were followed up to 10 days. In the context of this investigation, we chose to define the stability of a phantom as the period over which the mean concentration of the chromophore of interest (either water or hemoglobin) remains within 10% of its initial estimate.

## 3 Results

### 3.1 Varying Water Absorption

**Fig. 1:**
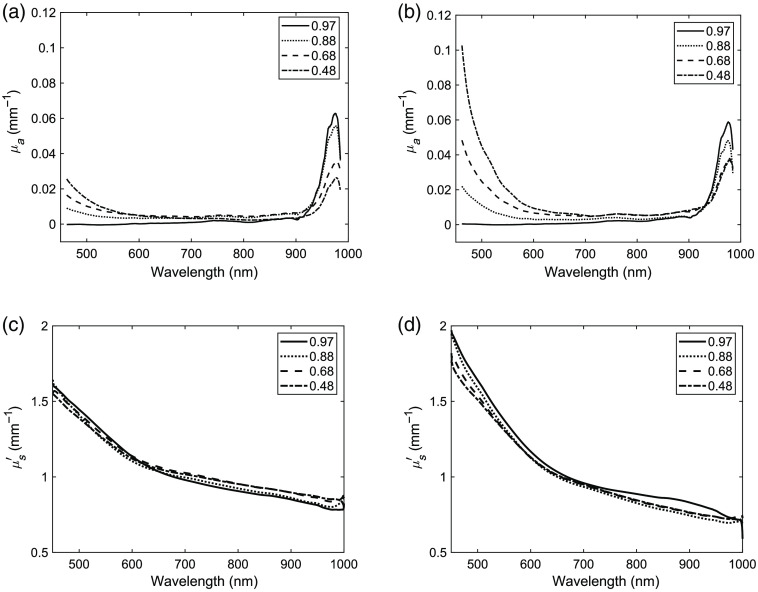
Absorption and reduced scattering spectra for phantoms including 10% Intralipid^®^ 20% and four different total water fractions ranging from 0.97 to 0.48. (a) Absorption coefficient store-bought gelatin, (b) absorption coefficient research grade gelatin, (c) reduced scattering coefficient store-bought gelatin, and (d) reduced scattering coefficient research grade gelatin.

Figure 1 shows representative absorption and reduced scattering spectra for phantoms with different total water fractions, described in Table 1, for both types of gelatins. The absorption spectra of the two types of gelatins mainly differed below 580 nm, where the research grade gelatin had stronger absorption than the store-bought gelatin. This was also observed visually, where phantoms including research grade gelatin having a stronger yellow color compared to those including store-bought gelatin. On average, the absorption peak at 970 nm scaled linearly with the water fractions of the phantoms for both types of gelatins. The reduced scattering coefficient spectra exhibited similar shapes, further indicating that the concentration of gelatin did not impact the expected power-law-like behavior of scattering in homogenous media with both Rayleigh and Mie Scattering contributions.^26^

**Fig. 2:**
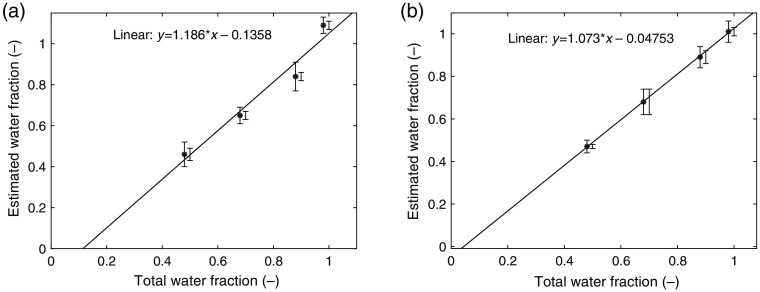
Estimated water fraction of the phantoms versus total water fraction: (a) store-bought gelatin and (b) research grade gelatin. The solid line shows the linear regression model for each gelatin. Error bars represent standard deviation based on multiple batches of each water fraction, and the error bars to the side represent standard deviation based on multiple measurements on each phantom (spatial variability).

Figure 2 shows the average estimated water fractions of the phantoms over multiple batches of phantoms and multiple measurements versus total water fraction of the phantoms. The estimated water fractions were obtained by fitting the absorption spectra of the phantoms estimated by SFDS for absorption of water and gelatin. The error bars represent the standard deviation based on multiple batches of phantoms, and the error bars to the side represent the spatial variability within each phantom and were calculated based on multiple measurements of each phantom. The fitted linear regression model using an unconstrained fit was y=1.186*x−0.1358, $R^{2}=0.96$ for the store-bought gelatin and y=1.073*x−0.04753, $R^{2}=0.99$ for the research grade gelatin. However, if the linear regression model was constrained, f(0)=0 (i.e., it forced the regression to present a water fraction of zero for “pure” gelatin), the linear regression model was y=1.022*x, $R^{2}=0.93$ for the store-bought gelatin and y=1.014*x, $R^{2}=0.99$ for the research grade gelatin.

After ∼2 days, the estimated water fraction concentration reduced on average −8.0% for both the store-bought and the research grade gelatins relative to its initial value. Beyond 2 days, however, the phantoms remained stable up to 10 days, and the estimated water fraction changed on average 0.01 (1%) during that time period.

### 3.2 Hemoglobin Phantoms

**Fig. 3:**
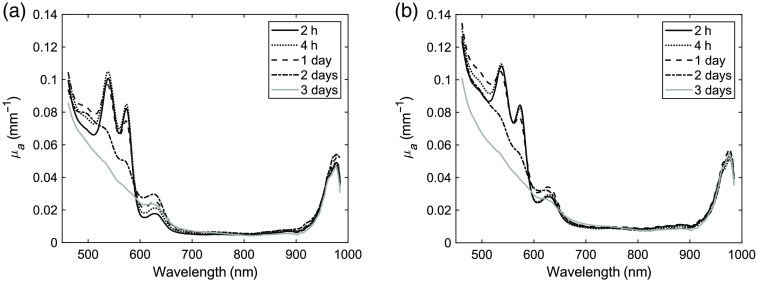
Changes in absorption spectra over time for phantoms including ferrous stabilized hemoglobin: (a) store-bought gelatin and (b) research grade gelatin.

Figure 3 shows the absorption spectra of the phantoms including ferrous stabilized human hemoglobin. Both phantoms showed a distinct peak at 630 nm, which corresponds to absorption features of methemoglobin (MetHb), and the absorption at 630 nm continued to increase up to day 2. Furthermore, a reduction was visible in the 520- to 590-nm wavelength range after 2 days for both types of gelatins, which corresponds to absorption features of oxygenated hemoglobin (${\mathrm{HbO}}_{2}$). This reduction continued to decrease at day 3 where oxygenated hemoglobin characteristics were no longer visible in the absorption spectra. These changes in absorption due to changes in oxygenated hemoglobin and methemoglobin could also be seen in the fitting parameter when fitting the absorption spectra for ${\mathrm{HbO}}_{2}$, deoxygenated hemoglobin (Hb), methemoglobin, and water (Table 2). The error values provided in this table (and in Table 3) are based on error estimations from the spectral fitting using a linear least squares model, e.g., $\chi^{2}$, as these estimations were larger than the standard deviation from multiple measurements, hence the more conservative metric.

**Table 2:** Concentrations of the fitted parameters of the two phantoms including ferrous stabilized hemoglobin when fitting for oxygenated hemoglobin, deoxygenated hemoglobin, methemoglobin, gelatin, and water.

	Store-bought gelatin				Research grade gelatin			
	${\mathrm{HbO}}_{2}$ (μM)	Hb (μM)	MetHb (μM)	Water (—)	${\mathrm{HbO}}_{2}$ (μM)	Hb (μM)	MetHb (μM)	Water (—)
2 h	2.07 ± 0.26	0 ± 0.23	1.10 ± 0.08	0.80 ± 0.07	1.72 ± 0.28	0 ± 0.26	1.49 ± 0.09	0.78 ± 0.08
4 h	1.98 ± 0.25	0 ± 0.23	1.26 ± 0.08	0.78 ± 0.07	1.79 ± 0.32	0 ± 0.29	1.70 ± 0.10	0.71 ± 0.08
1 day	1.41 ± 0.27	0 ± 0.25	1.59 ± 0.09	0.84 ± 0.08	1.25 ± 0.33	0 ± 0.30	1.96 ± 0.10	0.79 ± 0.10
2 days	0.28 ± 0.24	0 ± 0.22	1.83 ± 0.08	0.88 ± 0.10	0.58 ± 0.37	0 ± 0.34	1.90 ± 0.12	0.71 ± 0.11

**Table 3:** Concentrations of the fitted parameters of the two phantoms including whole blood when fitting for oxygenated hemoglobin, deoxygenated hemoglobin, methemoglobin, gelatin, and water.

	Store-bought gelatin				Research grade gelatin			
	${\mathrm{HbO}}_{2}$ (μM)	Hb (μM)	MetHb (μM)	Water (—)	${\mathrm{HbO}}_{2}$ (μM)	Hb (μM)	MetHb (μM)	Water (—)
2 h	3.71 ± 0.31	0 ± 0.28	0.52 ± 0.10	0.63 ± 0.06	4.24 ± 0.39	0 ± 0.36	0.32 ± 0.12	0.46 ± 0.07
4 h	3.65 ± 0.30	0 ± 0.27	0.53 ± 0.09	0.56 ± 0.05	4.37 ± 0.43	0 ± 0.39	0.37 ± 0.14	0.52 ± 0.08
1 day	3.37 ± 0.31	0 ± 0.28	0.60 ± 0.10	0.60 ± 0.07	4.03 ± 0.40	0 ± 0.37	0.45 ± 0.13	0.43 ± 0.07
2 days	3.28 ± 0.29	0 ± 0.26	0.60 ± 0.09	0.62 ± 0.08	4.09 ± 0.44	0 ± 0.40	0.55 ± 0.14	0.48 ± 0.09
3 days	2.90 ± 0.29	0 ± 0.26	0.87 ± 0.09	0.65 ± 0.07	2.31 ± 0.45	0 ± 0.41	1.06 ± 0.14	0.53 ± 0.09

**Fig. 4:**
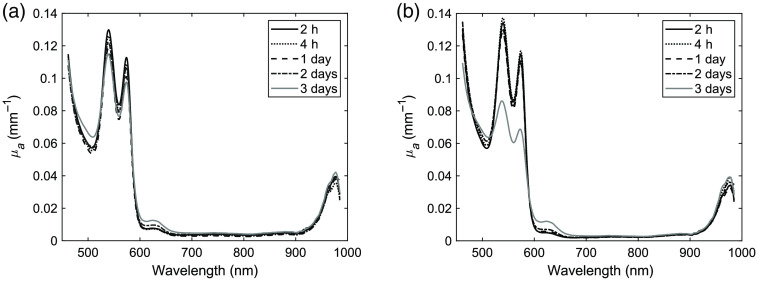
Changes in absorption spectra over time for phantoms including whole blood: (a) store-bought gelatin and (b) research grade gelatin.

Finally, Fig. 4 shows the absorption spectra for the phantoms including whole human blood for the two types of gelatins. Both phantoms had a small peak around 630 nm at 2 h, which increased slightly at day 3. Using the store-bought gelatin, the phantom with whole blood kept stable for 3 days. However, using the research grade gelatin, there was a noticeable reduction in the oxygenated hemoglobin peaks at day 3. At day 4, essentially no oxygenated hemoglobin characteristics were visible for either of the phantoms (not presented).

As presented in Table 3, the fitting parameters showed an overall higher amount of oxygenated hemoglobin and a lower amount of methemoglobin for the whole blood compared to the ferrous stabilized hemoglobin.

## 4 Discussion

In this study, we present a method for regulating the water and blood fraction in gelatin phantoms, so absorption and scattering in the model can be studied in detail. Once developed, the phantoms enable *in vitro* study of the details necessary for modeling interstitial fluid and analyte dynamics from biomedical optical techniques and *in vivo* microdialysis. Phantoms with different types of gelatins and gelatin-to-water fractions were characterized using SFDS. The estimated water fractions in the phantoms ranged from 1.09 to 0.46 for store-bought gelatin and from 1.01 to 0.47 for research grade gelatin. Ferrous stabilized hemoglobin or whole blood was added as absorbers in the phantoms, and the stability and hemoglobin absorption characteristics of phantoms with each absorber were compared. Ferrous stabilized hemoglobin had absorption features from methemoglobin at day 0 that continued to increase up to day 3, at which point the hemoglobin features were no longer visible in the spectra. Phantoms including whole blood kept stable up to 3 days using store-bought gelatin, whereas using research grade gelatin, the absorption characteristics from hemoglobin decreased after day 2. Although these gelatin phantoms may not have the long-term stability of PDMS phantoms, the hydrophilic nature of this media does present unique opportunities to incorporate water-based chromophores, such as hemoglobin and potentially others commonly found within *in vivo* tissues, while also independently varying the water fraction. Though not explored in this investigation, these phantoms also present the opportunity to design and incorporate distinct structures and layers into these phantoms given their solid to semisolid states. Epidermal layers to mimic skin pigmentation could be introduced to these phantoms in addition to underlying water and hemoglobin content.^21^

The ratio of gelatin-to-water affected the solidification rate of the phantoms. Phantoms including the lowest amount of gelatin never got fully solid and could not be removed from the plastic beakers. Even so, the phantoms were solid enough to enable stacking of layers on top of them, which makes it possible to create multilayer phantoms. At the other end, the phantoms including the highest amount of gelatin solidified at a high speed, and the process to remove the air bubbles resulting from the mixing was not always successful before the phantom had become solid.

The homogeneity of the phantoms was satisfactory based on the standard deviations in the estimated water fractions from the multiple measurements, presented in Fig. 2, and based on visual inspection of the phantoms. In the solidification process of the phantoms including 10% gelatin, there was a slight layering effect and a separation in concentration of lipid droplets, with the bottom of the phantom appearing to have a reduced concentration of lipid droplets and the top layer an increased concentration. This effect was reported previously by Puxiang et al.,^27^ who attributed it to the mixing temperature and the inclusion of gelatin causing the lipid droplets to aggregate and coalesce. By repeatedly mixing the phantoms carefully during the solidification process, the layering effect was not visible in the phantoms after 2 h when the phantoms were completely solid.

We found a linear relation between total and estimated water fractions for both gelatin types. The regression line deviated slightly from crossing (0, 0), especially for the store-bought gelatin. This deviation could originate from the slight overestimation in water concentration for the phantoms with the highest concentration of water (98%). Since there were no constraints in the fitting algorithm when fitting for water and gelatin, the estimated water fraction could result in values above 100%. In the case of the store-bought gelatin, the mean measured water fraction for 98% case was estimated to be 109%. When excluding the overestimated data point from the highest concentration of water, the regression line crossed (0, 0) for the store-bought gelatin ($R^{2}>0.999$, $\mathrm{RMSE}=10^{-16}$) using an unconstrained fit.

Different gelatin types affect the optical properties of the phantom differently. The research grade gelatin had absorption features at 450 nm, which was not significantly visible for the store-bought gelatin, similar to what has been reported elsewhere.^28^ This was also visible when inspecting the phantoms based on the research grade gelatin with the naked eye, where the phantoms with the highest ratio of gelatin had a strong yellow appearance. Furthermore, combining the research grade gelatin with whole blood, there was a faster breakdown of hemoglobin compared with the store-bought gelatin. This could possibly be due to the acidity of the research grade gelatin, where the research grade gelatin exhibited a slightly higher acidity to that of the store-bought gelatin (i.e., pH of 6.1 versus 6.3 at a gelatin fraction of 20%).

Both ferrous stabilized hemoglobin and whole blood produced distinct hemoglobin characteristics in the phantoms. Methemoglobin was present already at day 0 for the ferrous stabilized hemoglobin. This was expected due to the auto-oxidation of the heme group in the powder, where some methemoglobin is present already from the start in the powder. The auto-oxidation of the heme group was also visible over time as the fraction of methemoglobin increased in the phantoms including ferrous stabilized hemoglobin. Furthermore, powdered hemoglobin is expensive, and since wide field imaging techniques require large-volume phantoms to meet semi-infinite conditions, the cost of such phantoms is high. With the whole blood, there was basically no methemoglobin present at day 0, and the increase was minor during the first 2 days. Also the stability of the phantoms including whole blood was longer than that of the phantoms including ferrous stabilized hemoglobin. Although whole human blood can be a good alternative as a phantom absorber, it requires complicated sample preparation with safety protocols due to the potential risk for blood-borne pathogens and storage. Though ferrous stabilized hemoglobin has relatively poor stability and contamination from methemoglobin, there are still some advantageous features that may make it worth consideration. Unlike whole blood, ferrous stabilized hemoglobin can be directly ordered in a biologically purified form, significantly reducing risks from blood-borne pathogens. It also does not require access to local donors and clinical centers, which may be restricted by one’s location or the localities’ immediate health and safety mandates. From this investigation, however, there are clear and limiting considerations that must be made to utilize it as a proxy for *in vivo* human hemoglobin, namely, it must be used within 1 day of phantom fabrication and all interpretations of the spectral data must also account for the methemoglobin present.

We view the more sophisticated bench model opportunity given by the new phantoms as a potentially valuable addition to available techniques in the modeling of microdialysis data with the potential for extension from blood and water normalization to the issue of tissue tortuosity, the third major component influencing microdialysis data.^29^ The phantoms can contribute to our knowledge of the behavior of small molecules, such as glucose, in a tissue and can be convenient tools for the more complicated issues around measurement of larger molecules such as cytokines.^30^^,^^31^

## 5 Conclusion

We have described the fabrication and evaluation of gelatin-based tissue simulating optical phantoms having independently controllable absorption properties. The phantoms are designed to mimic tissue oedema and inflammation and include adjustable absorption from hemoglobin and water. Different gelatin types and preparations of human hemoglobin were investigated with regard to their intrinsic optical properties and stability. These low-cost phantoms offer the possibility to incorporate other water-based chromophore and dyes and can be used as an adaptive platform to evaluate the ability of diffuse optics systems to estimate tissue hemoglobin and water fractions. The phantoms can also be fabricated as thin sheets to form multilayered structures with added absorption features.
